# Efficacy of Antiangiogenic Drugs in the Treatment of Diabetic Macular Edema: A Bayesian Network Analysis

**DOI:** 10.3389/fphar.2021.637667

**Published:** 2021-04-15

**Authors:** Xuexue Zhang, Yi Liu, Miaoran Wang, Qiuyan Li, Wantong Zhang, Rui Zhang, Yufei Wu

**Affiliations:** ^1^ Xiyuan Hospital, China Academy of Chinese Medical Sciences, Beijing, China; ^2^ China Academy of Chinese Medical Sciences, Beijing, China; ^3^ Chongqing Medical University, Chongqing, China; ^4^ Beijing University of Chinese Medicine, Beijing, China

**Keywords:** antiangiogenic drugs, diabetic macular edema, network meta-analysis, best-corrected visual acuity, central macular thickness, intraocular pressure

## Abstract

**Aims:** To compare the efficacy of five kinds of antiangiogenic drugs in the treatment of diabetic macular edema

**Methods:** A comprehensive search of seven databases without language restrictions includes PubMed, EMBASE, Web of Science, CBM, the Cochrane Library, CNKI, and WanFang date. All literature used was published before October 2020. Eligible randomized trials were screened for inclusion in this study, and Bayesian framework was used to perform a network meta-analysis (NMA). Data on the mean change of best-corrected visual acuity (BCVA), central macular thickness (CMT) and intraocular pressure (IOP) at 6 months were extracted.

**Results:** 25 randomized controlled trials (RCTs) that covered 2214 eyes, which received treatment of more than 3 months durations were included. In the pooled pair-wise meta-analysis, there was no statistically significant difference between all treatments. The same result was observed in the network meta-analysis with 0–37.82% Global I-squared. For BCVA at 6 months, conbercept and ranibizumab may be favorable than bevacizumab, aflibercept, triamcinolone acetonide and sham injections according to the ranking probabilities. As for CMT at 6 months, ranibizumab may be the most effective compared to bevacizumab, aflibercept and triamcinolone acetonide. In terms of IOP at 6 months, ranibizumab have better effect than bevacizumab, triamcinolone acetonide and sham injections. The results of sensitivity analysis also confirm it.

**Conclusion:** The analysis confirms that ranibizumab may be the most favorable for BCVA improvement and have a stronger efficacy in decreasing CMT and IOP than other drugs when taking all the indicators into consideration. This conclusion may provide clinical evidence to guide treatment decisions. However, more high-quality randomized controlled trials will be necessary to further confirm this.

## Introduction

Diabetic macular edema is one of the most common and serious complications of diabetes (Sayin et al., 2015). It is due to the unstable state of long-term high blood glucose levels that causes damage to the retinal vasculature, resulting in enhanced permeability of the retinal blood vessels and accumulation of fluid in the macular area, causing diabetic macular edema (Shin et al., 2014). Once diabetic macular edema occurs, it can significantly reduce vision. Data shows that about one-third of people with diabetes will develop retinopathy, and about 2.6% of blind syndromes worldwide can be contributed to diabetes (Leasher et al., 2016). With the development of a large number of randomized clinical trials, intravitreal injection has gradually replaced the method of grid laser photocoagulation, and has significantly improved the treatment efficacy of macular edema. Diabetic macular edema treatment guidelines issued by European Retina Specialist Association (EURETINA) in 2017 pointed out that anti-vascular growth factor is a safe and effective first-line treatment for diabetic macular edema (Schmidt-Erfurth et al., 2017).

There are many types of antiangiogenic drugs, the effectiveness of various drugs is different, and there is a lack of direct comparison evidence for these drugs, making it difficult to evaluate the effectiveness of multiple drugs. Moreover, there are few studies comparing the efficacy of drugs alone, and the number of included literatures is not enough. This is the first study which has estimated and compared the effectiveness of all triple antiangiogenic therapy that has been studied in randomized trials. Compared to same type meta-analysis, this study included a full range of antiangiogenic drugs and compared the differences in efficacy between single drugs. In addition, this study is of a higher standard, due to accurate experimental types of randomized trials, identifying interventions outside of laser interference, unity of follow-up time and outcome indicators. We aimed to evaluate the efficacy of five kinds of antiangiogenic drugs in the treatment of diabetic macular edema. This method can provide clinical evidence to guide treatment decisions.

## Materials and Methods

The study was conducted in accordance with the guidelines of the Cochrane Multiple Interventions Methods Group (Chaimani et al., 2017).

### Search Strategy

PubMed, EMBASE, Web of Science, CBM, the Cochrane Library, CNKI, and WanFang data were used to identify relevant studies published before October 2020. The search terms (diabetic macular edema) and (ranibizumab or bevacizumab or aflibercept or conbercept or pegaptanib or triamcinolone acetonide or dexamethasone or fluocinolone) and (randomized controlled trial or RCT or random) were used. All available literatures were considered for review. Manual search was also performed to ensure complete retrieval using references of key articles.

### Study Selection and Data Extraction

All investigators independently assessed all trials for eligibility and extracted data. After the initial extraction was completed, the results were checked by each investigator. In the case of disagreements, consensus was reached through discussion. The full text of all the articles were obtained and the same eligibility criteria was used to determine which, if any, to exclude at this stage. The follow-up time of each randomized trial, the average age and sex ratio of patients, grouping, intervention measures, and outcome indicators were recorded. Each reviewer independently read each article, assessed the completeness of the data extraction, and confirmed the quality rating.

### Eligibility Criteria

The included literature needs to meet the following criteria:1)Meets the relevant diagnostic criteria of type Ⅰ or Ⅱ diabetes complicated with diabetic macular edema; 2) Interventions can only be antiangiogenic drugs, including ranibizumab or bevacizumab or aflibercept or conbercept or triamcinolone acetonide or pegaptanib or dexamethasone or fluocinolone; 3) Outcome measures included BCVA or CMT or IOP more than 3 months; 4) Study design must be randomized controlled trials; 5) Only included patients who had not received laser treatment for at least 3 months before trials. Trials that included other drugs, trials that had incomplete or incalculable data, trials that did not have baseline data and trials that had left and right eyes compared to each other were excluded.

### Risk of Bias Within Individual Studies

Four independent reviewers (MR W, WT Z, R Z and YF W) reviewed all the included studies. Any disagreements were discussed and rechecked together. The Cochrane Collaboration using the Cochrane risk of bias tool of Review Manager were used for assessing risk of bias method in randomized controlled trials. After all the outcomes were evaluated, a summary of confidence examining the effectiveness of a comparison between ranibizumab and other drugs was created using the Grading of Recommendations Assessment, Development and Evaluation (GRADE) system (Nikolakopoulou et al., 2020).

### Clinical Endpoints

BCVA, CMT at 6 months were set as primary visual outcomes. In addition, IOP at 6 months was also used as important outcome indicator.

### Statistical Analysis

In order to make the results more reliable and reduce the heterogeneity between data, the mean changes before and after treatment were calculated. This was an intention-to-treat analysis based on random effect model. Summarized effect size was calculated as mean differences (MD). First, a direct meta-analysis of trials that compared different treatments was done. Then, the Bayesian network meta-analysis was conducted to compare between different treatment that no head-to-head trials.

Pairwise meta-analyses were conducted beforehand for every treatment comparison. Heterogeneity across individual studies was assessed by the Cochran Q test (Chi-squared) and Higgins I-squared inconsistency statistic. If there was no significant heterogeneity (*p* > 0.05, I-squared <50%), a fixed effects model was used. Otherwise, a random effects model was used. Given the low heterogeneity detected across all direct meta-analyses, fixed-effects estimates were reported.

This Bayesian network meta-analysis was done by R with gemtc package. Within the Bayesian hierarchical model frameworks, the number of chains was 3; thinning interval, 10; tuning iterations, 10,000; simulation iterations, 60,000; and initial values scaling, 2.5. Moreover, all the treatments were ranked based on the analysis of ranking probabilities.

Furthermore, the analysis at 6 months is star-shaped and does not contain a closed loop, so local inconsistency of the network cannot be assessed. Global inconsistency was judged by using the value of deviance information criterion (DIC) between the consistency and inconsistency model. If the DIC difference was within five, the data was generally considered consistent. To demonstrate the robustness of the results, a sensitivity analysis was performed. According to the follow-up time, the efficacy evaluation was brought forward to 3 months and later to 12 months, to see whether the conclusion was consistent with the follow-up time of 6 months. Inconsistency as well as statistical disagreement of direct and indirect evidence was assessed by using node-split method in sensitivity analysis. Moreover, a *p* > 0.05 was considered as no significant inconsistency. All data was analyzed by using R 3.6.3.

## Result

### Study Screening

**FIGURE 1:**
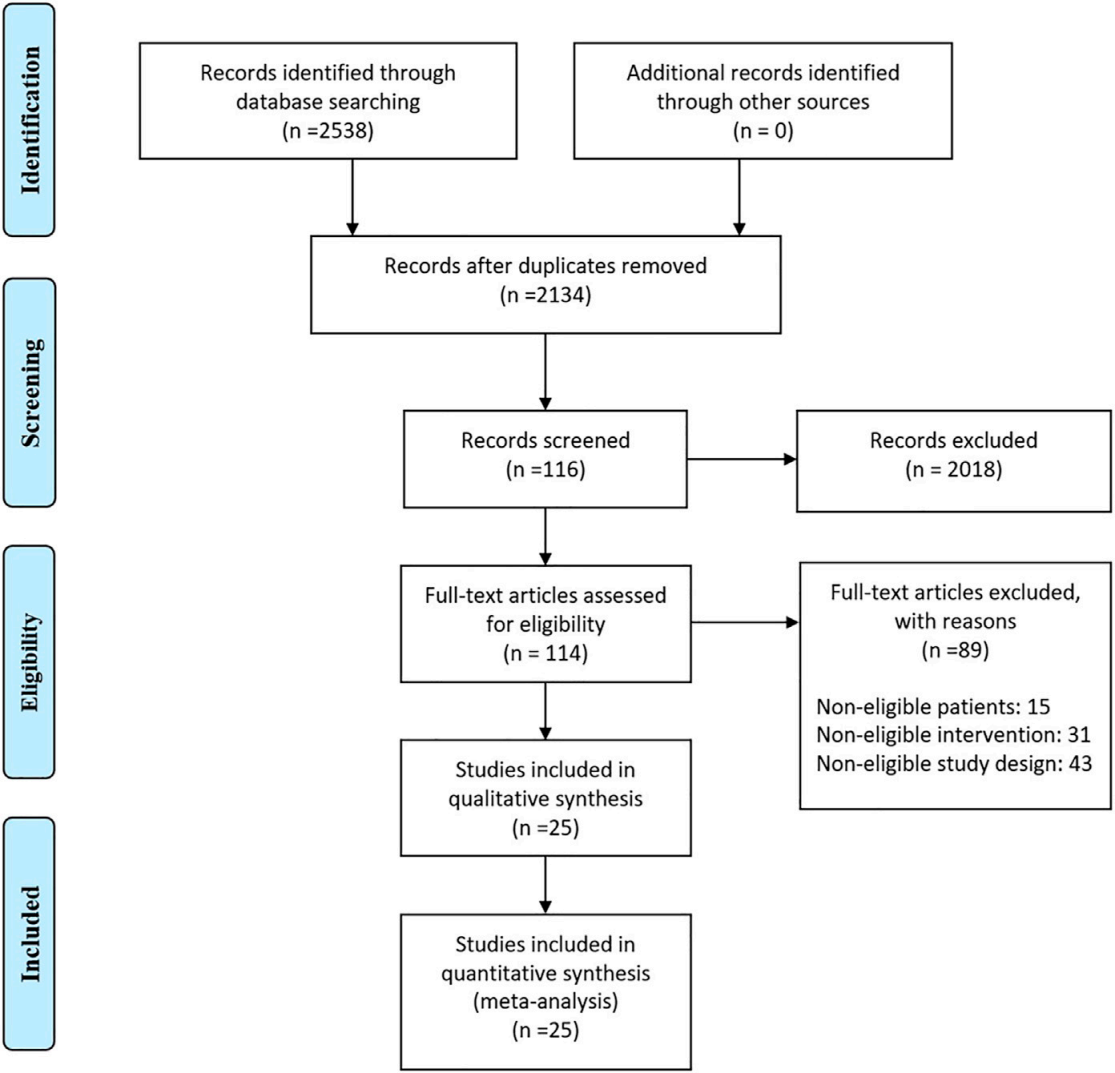
Flow chart indicating the selection process for this network meta-analysis.

Figure 1 shows the detailed steps of the literature selection process. Through searching the literature databases, 2538 relevant literatures were found, and 2424 duplications and unrelated literatures were excluded. Then, literatures were further selected by reading the full text, excluding substandard samples, substandard interventions and substandard research design, and finally included 25 articles.

### Study Characteristics and Network Geometry

**FIGURE 2:**
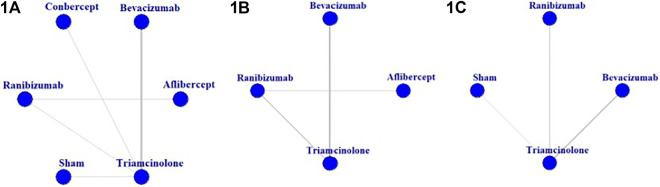
Network of eligible comparisons for the meta-analysis of **(1A)** best-corrected visual acuity at 6 months; **(1B)** central macular thickness at 6 months; **(1C)** intraocular pressure at 6 months.

In total, 25 trials were included in the network meta-analysis, the basic characteristics of them were presented in Table 1. A network of eligible comparisons for the multiple-treatment meta-analysis was constructed (Figure 2). Five treatments were analyzed: ranibizumab, bevacizumab, aflibercept, conbercept, triamcinolone acetonide. Most trials (22 of 25) were two-grouped studies (Jonas et al., 2006; Paccola et al., 2007; Wang et al., 2009; Ma, 2010; Massin et al., 2010; Marey and Ellakwa, 2011; Lim et al., 2012; Wan et al., 2013; Ekinci et al., 2014; Sonoda et al., 2014; Pan, 2016; Xue et al., 2016; Yan et al., 2016; Zhang et al., 2016b; Zhou, 2016; Fouda and Bahgat, 2017; Neto et al., 2017; Li et al., 2018; Ji et al., 2019; Ren et al., 2019; Schlingemann et al., 2019; Rodrigues et al., 2020) and the rest were three-grouped studies (Genentech, Inc., 2007; Baghi et al., 2017; Jabbarpoor et al., 2018). Overall, 2214 eyes were randomly assigned to one or two of the five antiangiogenic treatments or to the sham injection and were included in the network meta-analysis.

**TABLE 1:** The basic characteristics of the included literature.

Study id	Follow-up	Gender (male/female)	Mean age	Experimental group		Control group	
				No. of eyes	Intervention	No. of eyes	Intervention
Jonas et al. (2006)	24 weeks	NA	65.7 ± 7.5/68.8 ± 9.9	29	20 mg IVT	12	Sham injections
Genentech, Inc. (2007)	48 weeks	212/165	62.1 ± 9.6	T1:125 T2:125	T1:0.3 mg IVB T2:0.5 mg IVB	127	Sham injections
Paccola et al. (2007)	24 weeks	15/11	67.08 ± 4.67/65.58 ± 8.44	13	4 mg IVT	13	1.5 mg IVB
Wang et al. (2009)	48 weeks	7/17	52.4/51.6	12	4 mg IVT	12	Sham injections
Ma (2010)	24 weeks	22/33	60.34 ± 7.2	30	4 mg IVT	30	1.25 mg IVB
Massin et al. (2010)	48 weeks	81/70	63.6 ± 9.95	51	0.3 mg IVR	49	Sham injections
Marey and Ellakwa (2011)	12 weeks	53/37	57.64 ± 7.23	30	4 mg IVT	30	1.25 mg IVB
Lim et al. (2012)	48 weeks	50/55	60.4 ± 7.4	38	1.25 mg IVB	37	2 mg IVT
Wan et al. (2013)	12 weeks	24/30	55.8	30	0.5 mg IVR	30	4 mg IVT
Ekinci et al. (2014)	48 weeks	32/68	68 ± 9/65 ± 14	50	1.25 mg IVB	50	0.05 mg IVR
Sonoda et al. (2014)	12 weeks	34/17	59.2 ± 12.5/62.9 ± 11.4	25	4 mg IVT	26	1.25 mg IVB
Yan et al. (2016)	12 weeks	44/36	63.4 ± 9.6	40	0.5 mg IVR	40	4 mg IVT
Pan (2016)	24 weeks	44/34	66.5 ± 4.4/66.2 ± 4.2	39	0.05 ml IVR	39	0.05 mg IVT
Xue et al. (2016)	12 weeks	49/31	51.2 ± 3.7/51.9 ± 3.6	40	0.05 ml IVR	40	0.05 mg IVT
Zhang et al. (2016a)	24 weeks	33/27	62.4士3.4/63.1士2.9	30	0.05 ml IVR	30	0.05 mg IVT
Zhou (2016)	12 weeks	NA	NA	20	0.05 ml IVR	21	4 mg IVT
Neto et al. (2017)	24 weeks	43/34	NA	39	1.25 mg IVB	38	4 mg IVT
Fouda and Bahgat (2017)	48 weeks	NA	55.05 ± 4.7/56.64 ± 5.8	35	2 mg IVZ	35	0.5 mg IVR
Baghi et al. (2017)	12 weeks	51/72	63 ± 7	T1:42 T2:42	T1:2.5 mg IVZ T2:1.25 mg IVZ	39	1.25 mg IVB
Li et al. (2018)	48 weeks	55/47	58.0 ± 6.6/58.2 ± 6.7	51	0.05 ml IVR	51	0.05 mg IVT
Jabbarpoor et al. (2018)	48 weeks	51/72	63 ± 7	T1:42 T2:42	T1:2.5 mg IVZ T2:1.25 mg IVZ	39	1.25 mg IVB
Schlingemann et al. (2019)	24 weeks	101/65	63.9 ± 11.6/64.9 ± 11.6	84	1.25 mg IVB	82	0.5 mg IVR
Ji et al. (2019)	24 weeks	65/55	45.39 ± 4.22/45.87 ± 5.19	60	0.5 mg IVC	60	0.5 mg IVT
Ren et al. (2019)	12 weeks	37/41	57	30	0.5 mg IVR	30	2 mg IVT
Rodrigues et al. (2020)	24 weeks	39/26	60.7 ± 6.6/62.8 ± 8.2	32	1.25 mg IVB	28	1.20 mg IVT

Abbreviations: IVT, intravitreal triamcinolone acetonide; IVB, intravitreal bevacizumab; IVR, intravitreal ranibizumab; IVZ, intravitreal aflibercept; IVC, intravitreal conbercept; T, experimental group; C, control group; NA, no available.

The mean duration of the studies was 6 months (nine studies lasted 3 months, eleven lasted 6 months, and ten lasted 12 months or more), and the mean sample size was 43 eyes per group (minimum/maximum 12/127). In the sample included in the study, the gender ratio was balanced (males accounted for 55.0%) with an average age of about 60.62 years. In terms of clinical characteristics, most included studies recruited patients classified as having type Ⅰ or Ⅱ diabetes complicated with diabetic macular edema. The overall quality of studies was rated as good, despite some studies not disclosing details regarding randomize and allocation concealment.

### Risk of Bias and Quality Assessment

The quality of the studies included in this network meta-analysis is shown in Supplementary Figure S1. In relation to the random sequence generation, allocation concealment, blinding of participants and personnel and outcome assessment, one trial was rated as “high risk of bias” (1 of 25 trials), whereas for attrition bias and reporting bias, the majority of trials were rated as “low risk of bias”, because they reported the complete outcome data (24 and 25 trials respectively). Those rated at “unclear risk of bias” had issues relating to random sequence generation, and allocation concealment (13 and 21 trials, respectively) and masking of participants and personnel (16 trials/64.0%).

The results of the GRADE evaluation of interventions for ranibizumab were presented in Supplementary Table S1. All the reasons for downgrading were labeled. Due to the network analysis at 6 months is star-shaped and does not contain a closed loop, local inconsistency of the network cannot be assessed. Therefore, the level of evidence was all downgraded.

### Pair-Wise Meta-Analysis

In pooled pair-wise meta-analysis, there was no statistically significant difference between ranibizumab, bevacizumab, aflibercept, conbercept, triamcinolone acetonide and sham injections in BCVA at 6 months. As for CMT at 6 months, ranibizumab, bevacizumab, aflibercept and triamcinolone acetonide also did not show a statistically significant difference. In terms of IOP at 6 months, the same conclusion was observed in ranibizumab, bevacizumab, triamcinolone acetonide and sham injections.

## Network Meta-Analysis

### Best-Corrected Visual Acuity at 6 Months

**FIGURE 3:**
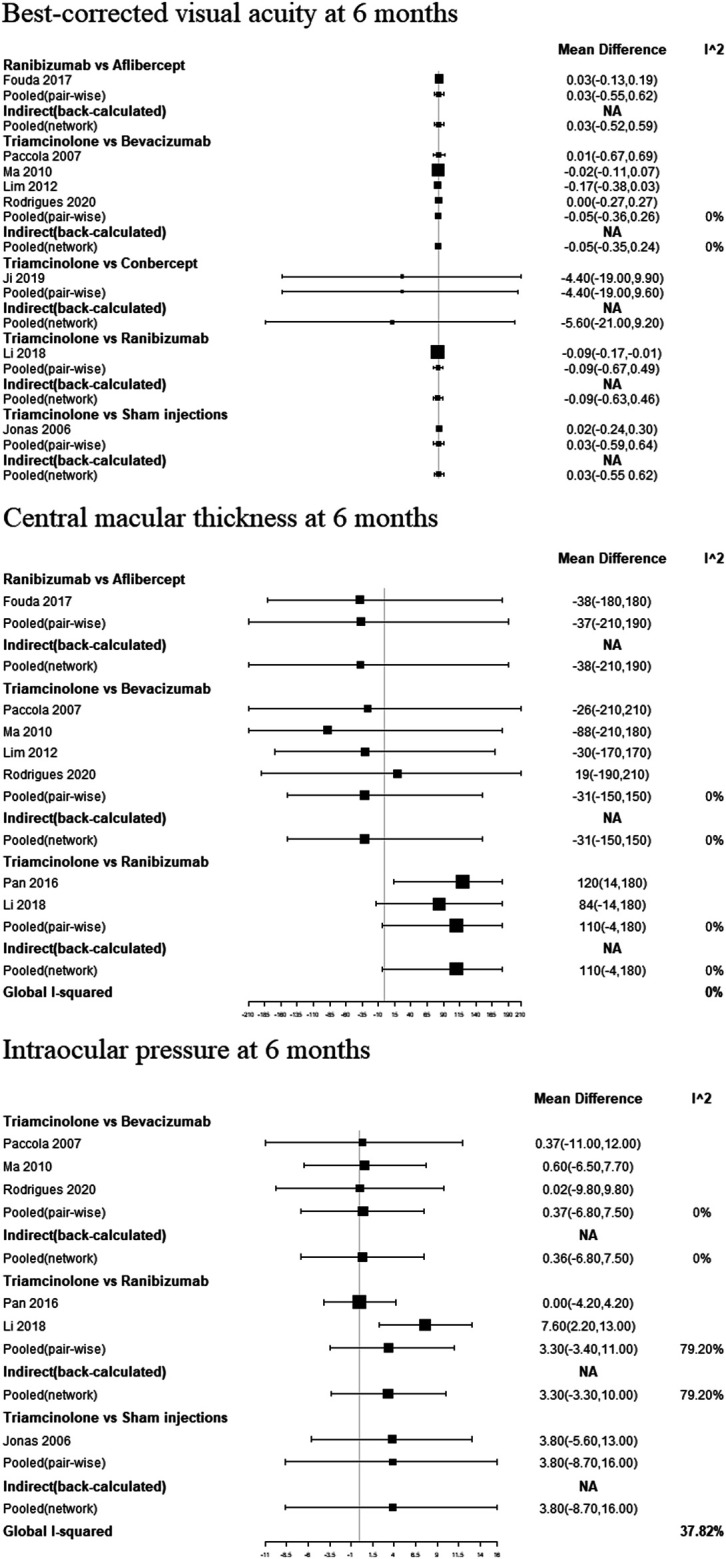
Pair-wise meta-analysis and network meta-analysis of different pharmacological interventions on effect of diabetic macular edema at 6 months.

**FIGURE 4:**
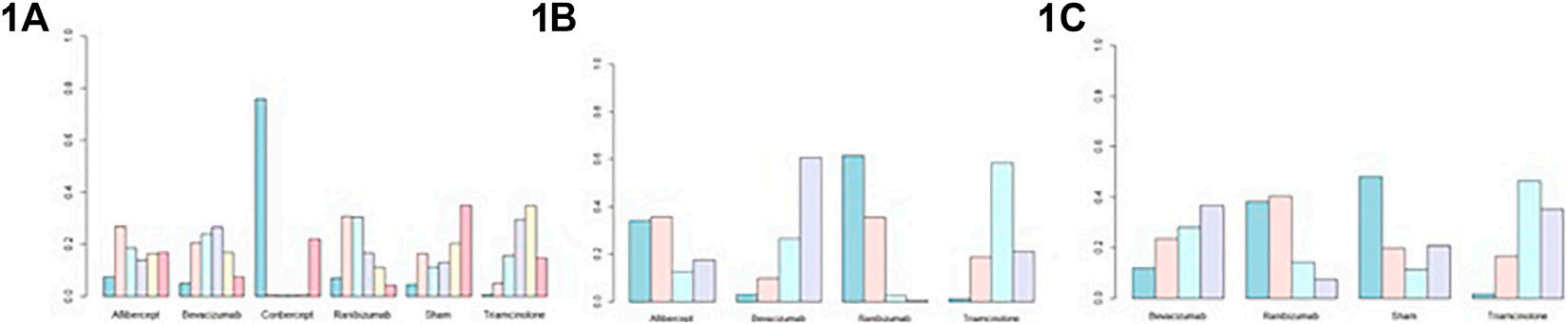
Probability plots of **(1A)** best-corrected visual acuity at 6 months; **(1B)** central macular thickness at 6 months; **(1C)** intraocular pressure at 6 months.

Figure 3 and Supplementary Table S2 shows the BCVA comparison results at 6 months. Non-significant results were found when comparing between all treatments. As for ranking the results, the efficacy of antiangiogenic drugs in order of most to least are as follows: conbercept, ranibizumab, aflibercept, triamcinolone acetonide, bevacizumab and sham injections (Figure 4).

### Central Macular Thickness at 6 Months

Figure 3 and Supplementary Table S2 show the CMT comparison results at 6 months. The NMA comparison using Bayesian framework indicated that ranibizumab, bevacizumab, aflibercept and triamcinolone acetonide also did not a show statistically significant difference. According to ranking probabilities, ranibizumab may decrease CMT more significantly than aflibercept, bevacizumab and triamcinolone acetonide, with aflibercept being probably the second most effective (Figure 4).

### Intraocular Pressure at 6 Months

IOP comparison results at 6 months are show in Figure 3 and Supplementary Table S2. There were no significant differences between pairs of treatment between ranibizumab, bevacizumab, sham injections and triamcinolone acetonide. As for ranking the results, ranibizumab may decrease IOP more significantly than bevacizumab, sham injections and triamcinolone acetonide (Figure 4). Meanwhile, bevacizumab may be the second most effective.

### Inconsistency Analysis

Global inconsistency of the network meta-analysis is summarized under consistency and inconsistency assumption (Supplementary Table S3). The difference of DIC between two models was no more than five, which means the results are generally considered consistent.

### Sensitivity Analysis

To demonstrate the robustness of our results, a sensitivity analysis was conducted by dividing different follow-up periods. After 3 months, both pooled pair-wise meta-analysis and network meta-analysis showed no statistically significant difference between all treatments (Supplementary Figure S2), with ranibizumab being the most favorable for BCVA improvement, decreasing CMT and IOP according to the ranking probabilities (Supplementary Figure S3). After 12 months, the same conclusion was observed in both pooled pair-wise meta-analysis and network meta-analysis (Supplementary Figure S4), with ranibizumab being the most favorable for BCVA improvement and decreasing CMT (Supplementary Figure S3). After sensitivity analysis, the main result revealed no significant change in NMA results, indicating the robustness and reliability of the statistical analysis.

## Discussion

This is the first study which has estimated and compared the effectiveness of all triple antiangiogenic therapy that has been studied in randomized trials. Compared to previous meta-analysis, this study included a full range of antiangiogenic drugs and compared the differences from three perspectives between single drugs. In addition, this study has a higher standard, due to accurate experimental types of randomized trials, identifying interventions outside of laser interference, unity of follow-up time and inclusion of trials published in non-English publications. A sufficient number of literatures, a total of 25 articles, was selected and included in this study.

According to the statistical results, it is shown that no matter which outcome indicator is compared or which follow-up period is observed, ranibizumab may have the best curative effect. A randomized controlled trial showed that the relative effect of intravitreous aflibercept, bevacizumab, or ranibizumab depended on baseline visual acuity, and that these drugs are equally effective in patients who initially had mild visual impairment (Wells et al., 2015), which is consistent with the comparison of the network meta-analysis. From a head-to-head trial, ranibizumab improves vision quickly and steadily compared with placebo and reduced the risk of long-term visual loss (Quan et al., 2012). Another study confirmed that ranibizumab significantly improved vision and is well tolerated by patients.

A recent review (Zhang et al., 2016a) published in 2016 that compared 21 trials (4703 eyes), suggested that intravitreal aflibercept is most favorable, with both BCVA improvement and CMT reduction within 12 months compared with other current therapies in the management of diabetic macular edema. In addition, another study (Pham et al., 2019) evaluated head-to-head randomized controlled trials comparing aflibercept, bevacizumab, and ranibizumab (or any combination of head-to-head comparisons) in adult patients with diabetic macular edema. The study found that patients with diabetic macular edema treated with aflibercept experienced significantly higher vision gain at 12 months than patients receiving ranibizumab or bevacizumab. This is slightly different from the conclusion reached in this study, which indicated aflibercept is second only to ranibizumab in both BCVA improvement and CMT reduction according to the ranking probabilities.

With respect to the efficacy of the various drugs, short-term and long-term efficacy were compared, and data from three periods (3, 6, and 12 months). As for the safety of antiangiogenic drugs in the treatment of diabetic macular edema, IOP was chosen to quantitatively evaluate this. Because the amount of data available regarding IOP at 12 months is limited, only data from 3 to 6 months were compared. There was no statistical difference in the efficacy of all drugs at 6 months.

This study has limitations to some extent, because the present literature is not comprehensive enough, random test sample size is not big enough, and it incorporated literature of variable quality. In addition, the research results were impacted by using selected trials from different regions, ages of the patients, and levels of medical treatment. These factors may have a potential impact on the results, although it was found that the heterogeneity of the study is not high. Moreover, measurements that are not easily affected by patients' baseline conditions, including changes in visual acuity, central macular thickness and intraocular pressure before and after treatment, thus the statistical analysis and conclusions are relatively reliable. 3, 6, and 12 months after the operation were chosen as times of result analysis. This choice was guided by the availability of various trial data, which also helped to compare the short-term and long-term effects of using the drugs.

This systematic review and network meta-analysis determined the efficacy of different anti-angiogenic drugs on diabetic macular edema. The analysis confirms that ranibizumab may be most favorable for BCVA improvement and have a stronger efficacy in decreasing CMT and IOP than using other drugs when taking all the indicators into consideration. This conclusion may help doctors evaluate the balance of pros and cons of various drugs and adjust their treatment accordingly.

## Data Availability

The original contributions presented in the study are included in the article/Supplementary Material, further inquiries can be directed to the corresponding authors.
