# Supplementary material for: Efficacy of Antiangiogenic Drugs in the Treatment of Diabetic Macular Edema: A Bayesian Network Analysis
Source: Front Pharmacol. 2021 Apr 15;12:637667. doi: 10.3389/fphar.2021.637667 (PMC8082725; doi:10.3389/fphar.2021.637667)

**LEGENDS FOR SUPPLEMENTARY TABLES**

Supplementary Table 1: Summary of confidence (GRADE approach) examining the effectiveness of a comparison between ranibizumab and other drugs.

Supplementary Table 2: League table describing clinical effectiveness of all treatments according to network meta-analysis: 1a) best-corrected visual acuity at 3 months; 1b) best-corrected visual acuity at 6 months; 1c) best-corrected visual acuity at 12 months; 2a) central macular thickness at 3 months; 2b) central macular thickness at 6 months; 2c) central macular thickness at 12 months; 3a) intraocular pressure at 3 months; intraocular pressure at 6 months.

Supplementary Table 3: A comparison of consistency and inconsistency model of: a) best-corrected visual acuity at 6 months; b) central macular thickness at 6 months; c) intraocular pressure at 6 months.

**LEGENDS FOR SUPPLEMENTARY FIGURES**

Supplementary Figure 1. Assessment of the methodological quality of the included studies.

Supplementary Figure 2. Pair-wise meta-analysis and network meta-analysis of different pharmacological interventions on effect of diabetic macular edema at 3 months.

Supplementary Figure 3. Probability plots of 1a) best-corrected visual acuity at 3 months; 1b) central macular thickness at 3 months; 1c) intraocular pressure at 3 months; 2a) best-corrected visual acuity at 12 months; 2b) central macular thickness at 12 months.

Supplementary Figure 4. Pair-wise meta-analysis and network meta-analysis of different pharmacological interventions on effect of diabetic macular edema at 12 months.

|  | Comparison | Mixed evidence | |
| --- | --- | --- | --- |
| Outcome indicator |  | MD (95%Cl) | Quality of evidence |
| Best-corrected visual acuity at 6 months | Aflibercept vs Ranibizumab | -0.03(-0.57, 0.53) | Very low ^ab^ |
|  | Ranibizumab vs Triamcinolone acetonide | 0.09(-0.44, 0.623) | Low ^a^ |
|  |  | Indirect evidence | |
|  |  | MD (95%Cl) | Quality of evidence |
|  | Bevacizumab vs Ranibizumab | -0.039(-0.65, 0.56) | Very low ^ab^ |
|  | Conbercept vs Ranibizumab | 4.70 (-8.30, 18.02) | Very low ^ab^ |
|  | Ranibizumab vs Sham injections | 0.12(-0.67, 0.89) | Very low ^ab^ |
|  |  | Mixed evidence | |
|  |  | MD (95%Cl) | Quality of evidence |
| Central macular thickness at 6 months | Aflibercept vs Ranibizumab | 38.25(-157.96, 232.52) | Very low ^ab^ |
|  | Ranibizumab vs Triamcinolone acetonide | -104.79(-216.50, 4.02) | Low ^a^ |
|  |  | Indirect evidence | |
|  |  | MD (95%Cl) | Quality of evidence |
|  | Bevacizumab vs Ranibizumab | 135.73(-22.31, 296.43) | Low ^a^ |
|  |  | Mixed evidence | |
|  |  | MD (95%Cl) | Quality of evidence |
| Intraocular pressure at 6 months | Ranibizumab vs Triamcinolone acetonide | -3.369 (-10.53, 3.24) | Very low ^ab^ |
|  |  | Indirect evidence | |
|  |  | MD (95%Cl) | Quality of evidence |
|  | Bevacizumab vs Ranibizumab | 3.033 (-6.64, 13.22) | Very low ^ab^ |
|  | Ranibizumab vs Sham injections | 0.464 (-14.01, 14.48) | Very low ^ab^ |

^a^ Incoherence; ^b^ Imprecision

1a)

| Aflibercept | -0.009  (-0.132, 0.109) | 0.002  (-0.142, 0.145) | -0.328  (-0.718, 0.070) | -0.039 (-0.181, 0.095) |
| --- | --- | --- | --- | --- |
| 0.009  (-0.109, 0.132) | Bevacizumab | 0.0107  (-0.121, 0.143) | -0.319 (-0.699, 0.067) | -0.031  (-0.131, 0.065) |
| -0.002  (-0.145, 0.142) | -0.011  (-0.143, 0.121) | Ranibizumab | -0.329 (-0.709, 0.061) | -0.041(-0.153, 0.064) |
| 0.328 (-0.070, 0.718) | 0.319  (-0.067, 0.699) | 0.329  (-0.061, 0.709) | Sham injections | 0.287  (-0.087, 0.654) |
| 0.0399  (-0.095, 0.181) | 0.0306  (-0.065, 0.131) | 0.0412 (-0.064, 0.153) | -0.287  (-0.654, 0.087) | Triamcinolone acetonide |

1b)

| Aflibercept | -0.010  (-0.841, 0.806) | 4.749 (-8.283, 18.069) | 0.0294  (-0.525, 0.573) | -0.092  (-1.041, 0.857) | -0.059 (-0.839, 0.701) |
| --- | --- | --- | --- | --- | --- |
| 0.010  (-0.806, 0.841) | Bevacizumab | 4.748  (-8.258, 18.043) | 0.0392  (-0.560, 0.647) | -0.082  (-0.727, 0.568) | -0.051(-0.343, 0.242) |
| -4.749 (-18.069, 8.283) | -4.748 (-18.043, 8.258) | Conbercept | -4.706  (-18.021, 8.302) | -4.819 (-18.144, 8.228) | -4.802 (-18.103, 8.203) |
| -0.029  (-0.573, 0.525) | -0.039  (-0.647, 0.560) | 4.706  (-8.302, 18.021) | Ranibizumab | -0.121  (-0.899, 0.667) | -0.089 (-0.627, 0.441) |
| 0.092  (-0.857, 1.041) | 0.082  (-0.568, 0.727) | 4.819 (-8.228, 18.144) | 0.121  (-0.667, 0.899) | Sham injections | 0.030  (-0.552, 0.602) |
| 0.0598  (-0.7018, 0.8398) | 0.051 (-0.242, 0.343) | 4.802  (-8.203, 18.103) | 0.089  (-0.441, 0.629) | -0.030  (-0.602, 0.552) | Triamcinolone acetonide |

1c)

| Aflibercept | -0.215  (-6.597, 5.456) | 0.735  (-6.215, 8.867) | -4.114  (-15.287, 4.131) | -0.695  (-8.793, 6.247) |
| --- | --- | --- | --- | --- |
| 0.215 (-5.456, 6.597) | Bevacizumab | 1.194  (-5.820, 9.665) | -3.641  (-14.123, 4.251) | -0.312  (-6.784, 5.315) |
| -0.735  (-8.867, 6.215) | -1.194 (-9.665, 5.820) | Ranibizumab | -5.355 (-14.849, 1.070) | -1.806 (-9.384, 4.199) |
| 4.114  (-4.131, 15.287) | 3.641 (-4.251, 14.123) | 5.355  (-1.070, 14.849) | Sham injections | 3.129  (-3.403, 11.899) |
| 0.695  (-6.247, 8.793) | 0.312  (-5.315, 6.784) | 1.806  (-4.199, 9.384) | -3.129 (-11.899, 3.403) | Triamcinolone acetonide |

2a)

| Aflibercept | 8.657  (-43.998, 63.152) | -24.442  (-119.317, 71.309) | -1.493  (-84.746, 81.386) |
| --- | --- | --- | --- |
| -8.657  (-63.152, 43.998) | Bevacizumab | -33.737  (-120.419, 54.389) | -10.249  (-80.344, 59.101) |
| 24.442  (-71.309, 119.317) | 33.737  (-54.389, 120.419) | Ranibizumab | 23.143 (-38.607, 85.157) |
| 1.493  (-81.386, 84.746) | 10.249  (-59.101, 80.344) | -23.143 (-85.157, 38.607) | Triamcinolone acetonide |

2b)

| Aflibercept | 97.399  (-153.203, 349.181) | -38.269  (-232.524, 157.963) | 65.598 (-153.929, 292.776) |
| --- | --- | --- | --- |
| -97.399 (-349.181, 153.203) | Bevacizumab | -135.731 (-296.427, 22.311) | -31.004 (-145.421, 84.918) |
| 38.269 (-157.963, 232.524) | 135.731  (-22.311, 296.427) | Ranibizumab | 104.799 (-4.022, 216.502) |
| -65.598  (-292.776, 153.929) | 31.004 (-84.918, 145.421) | -104.799  (-216.502, 4.022) | Triamcinolone acetonide |

2c)

| Aflibercept | 14.787  (-29.082, 60.099) | 11.527  (-106.481, 129.190) | 6.288  (-84.388, 99.721) |
| --- | --- | --- | --- |
| -14.787  (-60.099, 29.082) | Bevacizumab | -3.596  (-121.588, 115.532) | -8.482  (-93.587, 77.115) |
| -11.527  (-129.190, 106.481) | 3.596 (-115.532, 121.588) | Ranibizumab | -4.529  (-123.093, 111.287) |
| -6.288  (-99.721, 84.388) | 8.482  (-77.115, 93.587) | 4.529  (-111.287, 123.093) | Triamcinolone acetonide |

3a)

| Bevacizumab | -3.522  (-11.667, 4.923) | 0.577  (-4.594, 5.634) |
| --- | --- | --- |
| 3.522 (-4.923, 11.667) | Ranibizumab | 4.119 (-2.658, 10.461) |
| -0.577  (-5.634, 4.594) | -4.119  (-10.461, 2.658) | Triamcinolone acetonide |

3b)

| Bevacizumab | -3.033  (-13.221, 6.638) | -3.509 (-17.898, 10.887) | 0.369 (-6.788, 7.529) |
| --- | --- | --- | --- |
| 3.033  (-6.638, 13.221) | Ranibizumab | -0.464 (-14.484, 14.006) | 3.369  (-3.237, 10.528) |
| 3.509  (-10.887, 17.898) | 0.464  (-14.006, 14.484) | Sham | 3.864 (-8.717, 16.329) |
| -0.369  (-7.529, 6.788) | -3.369  (-10.528, 3.237) | -3.864  (-16.329, 8.717) | Triamcinolone acetonide |

a)

| Consistency model | | | Inconsistency model | | |
| --- | --- | --- | --- | --- | --- |
| DIC | Ratio | I^2 | DIC | Ratio | I^2 |
| 28.91960 | 0.9173 | 0% | 29.43417 | 0.9334 | 0% |

b)

| Consistency model | | | Inconsistency model | | |
| --- | --- | --- | --- | --- | --- |
| DIC | Ratio | I^2 | DIC | Ratio | I^2 |
| 22.12043 | 0.8070 | 0% | 22.24354 | 0.8111 | 0% |

c)

| Consistency model | | | Inconsistency model | | |
| --- | --- | --- | --- | --- | --- |
| DIC | Ratio | I^2 | DIC | Ratio | I^2 |
| 22.03413 | 0.9514 | 4% | 22.04712 | 0.9536 | 4% |


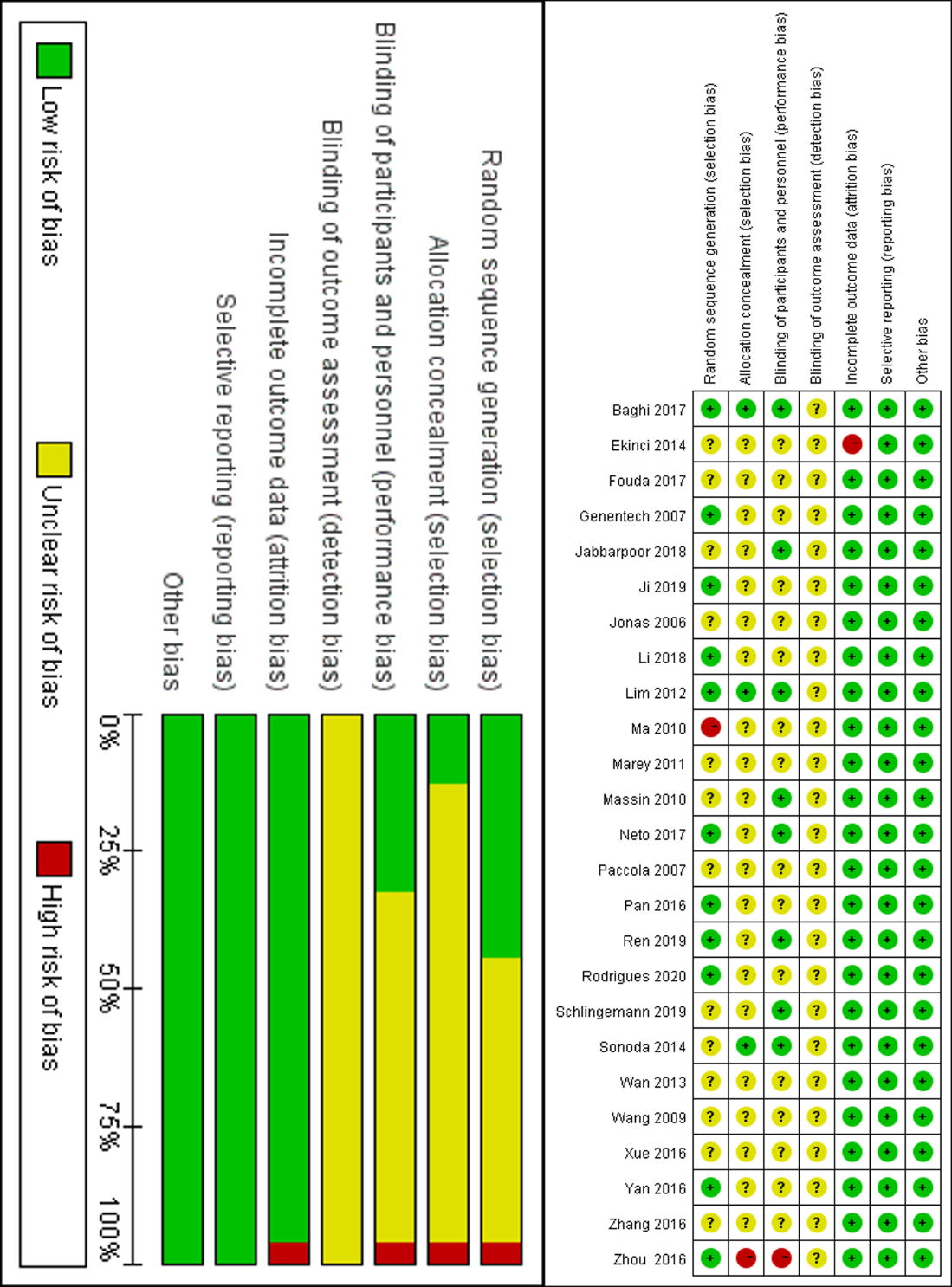


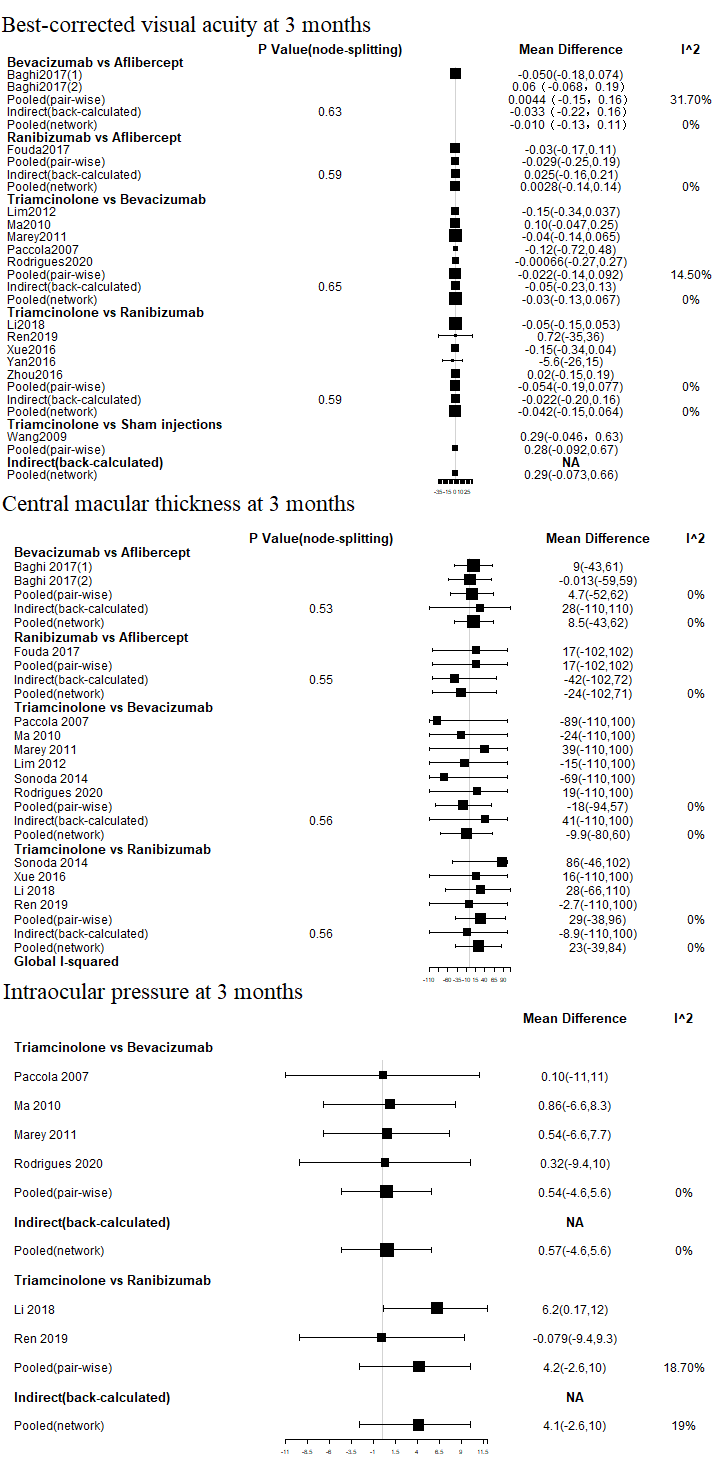


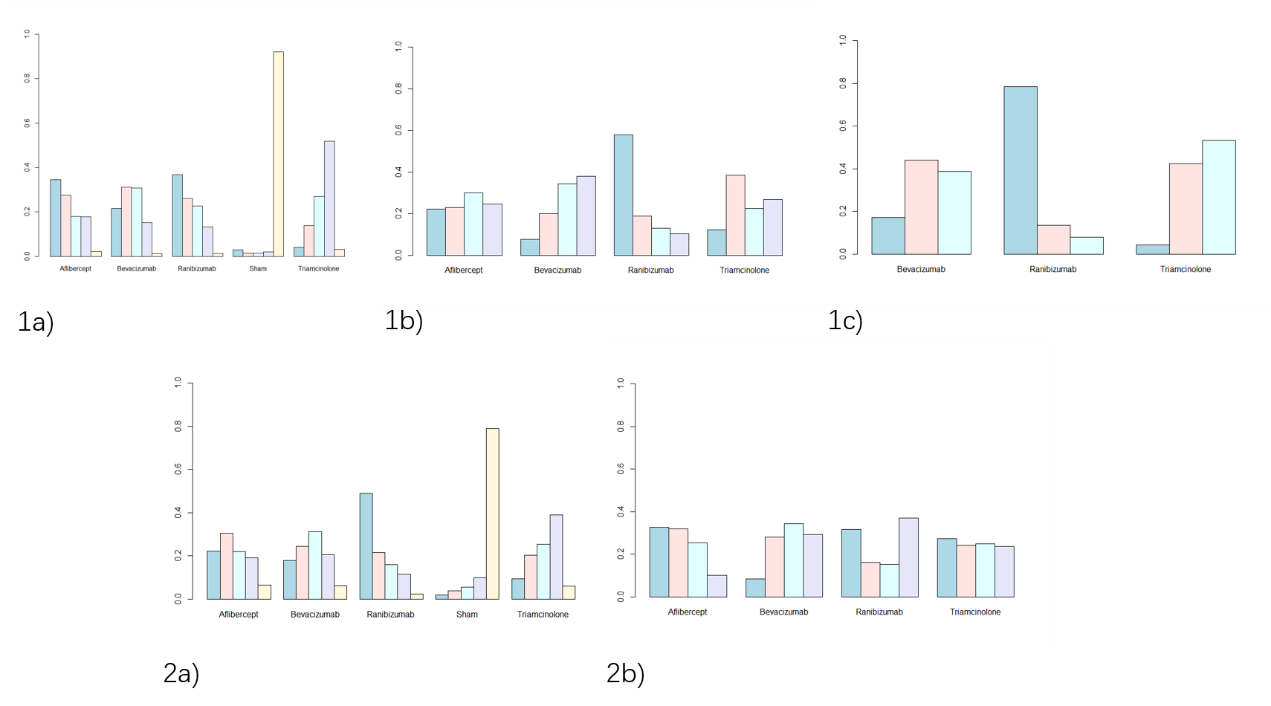


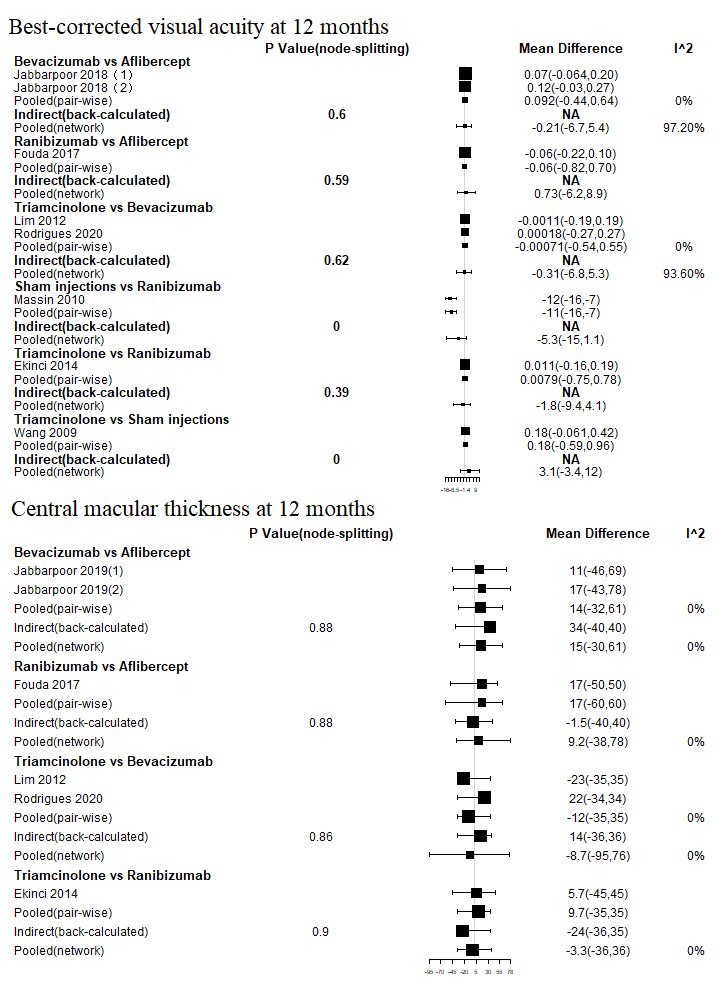

Supplement: Supplementary file 1 [file datasheet1.docx]
